# Conservative, Non-pharmacological Interventions for Pain Management in Juvenile Idiopathic Arthritis: A Systematic Review and Meta-Analysis of Randomized Controlled Trials

**DOI:** 10.7759/cureus.73295

**Published:** 2024-11-08

**Authors:** Lisa Musso-Daury, Tamara Pascual Fernández, Susana López-Ortiz, Mónica Pico De Las Heras, Enzo Emanuele, Simone Lista, Carmen Matey-Rodríguez, Alejandro Santos-Lozano

**Affiliations:** 1 Health Sciences, i+HeALTH Strategic Research Group, Miguel De Cervantes European University, Valladolid, ESP; 2 Health Sciences, Miguel De Cervantes European University, Valladolid, ESP; 3 Scientific Directorate, 2E Science, Robbio, ITA

**Keywords:** exercise, juvenile idiopathic arthritis, meta-analysis, non-pharmacological interventions, pain, randomized controlled trials, systematic review

## Abstract

We conducted a systematic review and meta-analysis of randomized controlled trials (RCTs) to assess the effectiveness of conservative, non-pharmacological interventions for chronic pain management in children and adolescents with juvenile idiopathic arthritis (JIA). A comprehensive search strategy was implemented across PubMed, PEDro, and Web of Science databases, utilizing predefined terms and strict inclusion and exclusion criteria. The initial search yielded 1,308 studies, which were subsequently narrowed to 65 relevant articles. Following a rigorous evaluation, 14 studies met the inclusion criteria for final review, with an average PEDro scale score of 6.1/10, indicating fair to good methodological quality. The included RCTs focused on various interventions, including physical exercise (five studies), hydrotherapy (three studies), orthoses (two studies), online cognitive behavior therapy for pain management (two studies), low-level laser therapy (one study), and video games (one study). A random-effects model meta-analysis was performed for interventions and outcome measures that were comparable across at least three RCTs. Physical exercise interventions met this criterion and were thus subjected to meta-analytic evaluation. The pooled analysis demonstrated a statistically significant beneficial effect of exercise interventions on chronic pain (mean difference (MD) = -1.37, 95% CI = -2.19 to -0.55, p < 0.01). Subgroup analyses further supported the efficacy of exercise compared to both other active interventions (MD = -1.37, 95% CI = -2.25 to -0.5, p < 0.01) and control conditions (MD = -1.69, 95% CI = -3.09 to -0.29, p = 0.02). These findings suggest that conservative, non-pharmacological interventions, particularly physical exercise, show promise as a component of a multidisciplinary pain management strategy for patients with JIA. While further high-quality research is needed to bolster the evidence base, our findings highlight the potential efficacy of integrating physical exercise interventions into comprehensive pain management strategies for this pediatric population.

## Introduction and background

Juvenile idiopathic arthritis (JIA) is typically defined as a heterogeneous group of chronic inflammatory arthropathies that manifest before 16 years of age and persist for a minimum of six weeks [1-3]. However, some organizations, such as the Pediatric Rheumatology International Trials Organization (PRINTO), extend this definition to include patients diagnosed before 18 years of age [4]. JIA encompasses various forms of persistent childhood arthritis, affecting not only joints but also extra-articular structures such as the eyes, skin, and internal organs, potentially leading to disability and, in rare cases, mortality [5]. As the most prevalent chronic rheumatological condition in pediatric populations, JIA affects approximately three million children and adolescents globally [6]. The estimated incidence in Caucasian children is 8.3 per 100,000, with a gender disparity of 10 per 100,000 in females and 5.7 per 100,000 in males [6].

The International League of Associations for Rheumatology (ILAR) has established a standardized classification system for JIA to facilitate consistent diagnosis and research across the global rheumatology community [3]. This system delineates seven distinct subtypes based on clinical manifestations and laboratory findings, i.e., systemic arthritis, oligoarthritis (persistent and extended), psoriatic arthritis, seropositive polyarthritis, seronegative polyarthritis, enthesitis-related arthritis, and undifferentiated arthritis [2,3]. The ILAR classification employs exclusion criteria to ensure mutual exclusivity among categories, thereby enhancing the homogeneity of patient cohorts for research purposes. However, it is important to note that this classification system is considered provisional and subject to refinement as our understanding of JIA’s molecular pathogenesis evolves [7].

The etiology of JIA remains elusive, posing significant challenges in establishing a definitive diagnosis [4,5]. However, current evidence suggests that genetic predisposition, particularly involving the human leukocyte antigen (HLA) system, along with dysregulation of the immune response, specifically the interleukins IL-1 and IL-6, and inflammatory processes, play a key role in the development of JIA [8]. Additionally, recent studies have highlighted the potential involvement of the microbiome in modulating immune function and contributing to disease onset [9]. These factors converge to induce chronic inflammation of the synovial membrane, leading to cartilage degradation, bone erosion, reduced joint space, and ultimately, pain and joint dysfunction [5]. Despite gaps in understanding the precise mechanisms, it is well-established that JIA can result in significant disability and functional impairment in affected children. The chronic pain associated with JIA, defined as discomfort persisting for more than three months, significantly affects patients' quality of life and daily functioning [2,10]. The ongoing pain often delays the performance of activities of daily living (ADLs), further impacting the child’s physical, emotional, and social development [10]. Beyond immediate well-being, chronic pain may also influence long-term outcomes, such as educational attainment, social integration, and overall life satisfaction [10].

The treatment paradigm for JIA has undergone a significant evolution over time, transitioning toward a comprehensive, multidisciplinary approach. This integrated strategy incorporates advanced surgical techniques, targeted pharmacological interventions, and conservative, non-pharmacological interventions. Recent advancements in pharmacological treatments, particularly the introduction of Janus Kinase (JAK) inhibitors, have shown significant promise [1]. However, it is crucial to note that the efficacy of JAK inhibitors may vary across different JIA subtypes, necessitating a tailored approach to treatment selection [10]. Accordingly, the therapeutic landscape for JIA currently includes a range of conservative, non-pharmacological interventions - including physiotherapy, psychological support, occupational therapies, and structured physical exercise programs. These approaches aim to manage pain, enhance functional capacity, and improve overall quality of life. While traditional surgical and pharmacological interventions often demonstrate more rapid and pronounced short-term efficacy, conservative, non-pharmacological treatments offer distinct advantages, including lower risk profile, potential for long-term benefits, and a holistic approach to patient care. It is also important to recognize that conservative therapies typically require consistent, long-term application to achieve optimal outcomes. This extended treatment duration allows for gradual improvement and adaptation, potentially leading to more sustainable results [11]. In addition, conservative treatments for JIA demonstrate a high degree of compatibility, enabling the integration of various modalities to create tailored therapeutic regimens [12,13]. This flexibility allows clinicians to address the diverse needs of individual patients, potentially enhancing treatment efficacy and patient outcomes [14]. Although conservative, non-pharmacological therapies are widely utilized in the management of JIA, there is a significant lack of comprehensive evaluations of these treatments in the literature [15-17]. To address this knowledge gap, this systematic review and meta-analysis of randomized controlled trials (RCTs) aims to 1) identify the primary conservative, non-pharmacological options for managing chronic pain in children with JIA, 2) describe the characteristics and implementation of these treatment approaches, and 3) evaluate the effectiveness of these interventions in alleviating chronic pain associated with JIA in pediatric patients. By synthesizing the available evidence, this study seeks to enhance our understanding of conservative approaches in JIA care and potentially guide future research directions in this critical area of pediatric rheumatology.

## Review

Materials and methods

This work adheres to the PRISMA guidelines for systematic reviews and meta-analyses [18].

Data Sources and Search Strategies

Two authors (LM-D, CM-R) independently conducted a comprehensive literature search in PubMed, Web of Science, and Physiotherapy Evidence Database (PEDro) databases for RCTs published in English, French, or Spanish up to September 22, 2023. The search strategy employed Medical Subject Headings terms and free-text keywords to ensure comprehensive coverage of conservative, non-pharmacological options for managing chronic pain in children with JIA. The following search terms were used: "Juvenile Arthritis" AND "Child" AND "Adolescent" AND "Chronic Pain" AND "Pain Management" NOT "Invasive" NOT "Oncology" NOT "Pharma*." To supplement the electronic database search, we conducted a manual review of reference lists from relevant publications to identify additional eligible studies. Two authors (LM-D, CM-R) independently evaluated the full texts of potentially relevant articles. In cases of disagreement, a third author (AS-L) was consulted to reach a consensus.

Study Selection

The inclusion criteria for RCTs in the systematic review were as follows: participants had to be aged from birth to 21 years and diagnosed with JIA for at least three months, experiencing chronic musculoskeletal pain that had persisted for more than three months. Only RCTs evaluating conservative, non-pharmacological treatments with chronic pain as an outcome measure were included, and no restrictions on the publication period were applied. Studies investigating surgical, pharmacologic, or invasive interventions were excluded, as were RCTs dealing with the prevention of pain associated with JIA.

Data Extraction

Two authors (LM-D and CM-R) independently performed the data extraction process using a standardized Excel spreadsheet. Any discrepancies in the extracted data were resolved through consultation with a third author (AS-L). From each included study, we extracted the following information: 1) bibliographic data; 2) sample characteristics, such as the age range of participants and the specific subtypes of JIA investigated; 3) intervention details, including the type of treatment, frequency and volume of sessions, duration of each session, and length of follow-up; and 4) pre- and post-intervention outcome measures, reported as mean values with standard deviations (SD) when available.

Methodological Quality

The methodological quality of the studies included in the systematic review was assessed using the PEDro scale [19]. This widely accepted tool, specifically designed for evaluating the quality of RCTs, consists of 11 items that assess key methodological aspects such as randomization, blinding, and attrition. Each item is scored as either present (1) or absent (0), with a maximum possible score of 10 points (the first item, eligibility criteria, is not included in the total score). Higher scores indicate better methodological quality. The PEDro scale has been demonstrated to have good reliability and validity in assessing the quality of RCTs in the field of physiotherapy and rehabilitation [19].

Meta-analysis

A random-effects model meta-analysis was conducted when a minimum of three RCTs employed comparable interventions and outcome measures [20,21]. Studies were included if they reported the mean effect of conservative treatments (defined as the difference between post-treatment and baseline data) or provided sufficient data to calculate this effect. The meta-analysis weighted each study based on the SD of variables and sample size. Publication bias was assessed using Begg’s test, while heterogeneity across studies was evaluated using the I2 statistic. A significance level of 0.05 was applied. All analyses were conducted using MIX 2.0 Pro for Excel software [22].

Results

**Figure 1:**
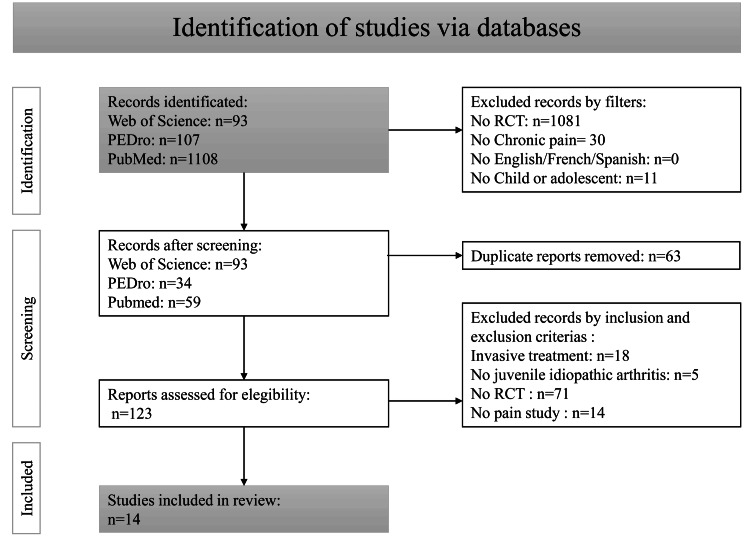
Flow diagram illustrating the study selection process

Our systematic search initially yielded 1,308 records. After screening titles and abstracts, we excluded 1,185 studies that did not meet our predefined criteria. We retrieved and assessed the full texts of the remaining 123 articles for eligibility. Following a comprehensive review, 14 RCTs satisfied all eligibility criteria and were included in the final analysis. These studies encompassed a total of 950 pediatric participants, with 508 children allocated to the experimental groups. Figure 1 illustrates the study selection process using a flow diagram.

Study Characteristics

The 14 included RCTs demonstrated a mean score of 6.1 on the PEDro scale, indicating fair to good methodological quality. Specifically, four studies were rated 8/10, five studies received a score of 7/10, and one study was rated 6/10 [23-32]. Additionally, three studies scored 5/10, and one study received a rating of 4/10 [33-36]. The 14 RCTs employed various interventions to alleviate pain in children with JIA, including online treatments, video games, low-level laser therapy, orthoses, hydrotherapy, and physical exercise interventions [23-36].

Patient Characteristics

The sample sizes in the reviewed RCTs exhibited substantial heterogeneity. At the lower end of the spectrum, Baydogan et al. and Elnaggar et al. conducted studies with the smallest cohorts (n = 30) [27,36]. In contrast, Conelly et al. executed the largest trial, encompassing 289 participants [26]. The mean sample size across studies was 68 participants, with a median of 25. Participant ages ranged from four years old to 21 years old [25,35]. The RCTs encompassed a diverse range of JIA subtypes and disease severities. Several studies - including those by Coda et al., Conelly et al., Armbrust et al., and Epps et al. - included all JIA subtypes in their analyses [25,26,30,31]. Other researchers focused on specific JIA categories. Specifically, Baydogan et al. included polyarticular (rheumatoid factor positive and negative), oligoarticular, and psoriatic arthritis [36]. Elnaggar et al. restricted their low-level laser therapy study to children with polyarticular JIA affecting both knees [27]. Perez Ramirez et al. focused on polyarticular, undifferentiated, and oligoarticular subtypes [33]. Mendonca et al. included oligoarticular, polyarticular (rheumatoid factor positive), and systemic JIA [23]. Arman et al. studied oligoarticular and polyarticular forms with upper limb joint involvement [34]. Tarakci et al. included polyarticular, oligoarticular, systemic, and psoriatic arthritis [28]. Powell et al. examined enthesitis-related, polyarticular, oligoarticular, and systemic JIA [29]. Sandstedt et al. investigated polyarticular, oligoarticular, enthesitis-related, and psoriatic arthritis [35]. Notably, Azab et al. and Elnaggar et al. did not specify the JIA subtypes in their studies [24,32]. Despite the variability in inclusion criteria, all included RCTs recruited patients with polyarticular JIA (Table 1).

**Table 1:** Summary of the 14 included studies CG, control group; EG, experimental group; N/A, not available; O, oligoarticular (extended or persistent); P, polyarticular; ER, enthesitis-related; PR, psoriasis-related; S, systemic; U, undifferentiated

Authors	JIA type	Age range	Groups, n, mean age, % females
Armbrust et al. (2017) [31]	O (extended or persistent); P RF (+); ER; PR; S	8 to 13 years	EG, n = 28, 9.7 (8.7-11.3), 75%
			CG, n = 21, 10.2 (9.0-10.8), 57%
Conelly et al. (2019) [26]	O (extended or persistent); P (RF (+), RF (-), or RF unknown) ER; PR; S; U	12 to 18 years	EG, n = 144, 14.6 ± 1.8, 68%,
			CG, n = 145, 14.5 ± 1.7, 77%
Arman et al. (2019) [34]	P; O	6 to 18 years	EG, n = 25, 12.36 ± 2.98, 84%
			CG, n = 25, 13.16 ± 3.35, 84%
Elnaggar et al. (2022) [32]	Do not specify subgroups	8 to 16 years	EG, n = 26, 11.6 ± 2.7, 34.6%
			CG, n = 27, 12.5 ± 2.3, 40.7%
Powell et al. (2005) [29]	P; O; ER; S	5 to 19 years	EG1, n = 15, 12.14 ± 3.32, 86.7%
			EG2, n = 12, 12.17 ± 3.04, 66.7%
			EG3, n = 13, 13.77 ± 4.55, 69.2%
Coda et al. (2014) [30]	O; P (RF (+), RF (-)); ER; S; U	5 to 18 years	EG, n = 31, 10.64 ± 3.84, 71%
			CG, n = 29, 11.17 ± 3.5, 79.3%
Perez Ramirez et al. (2019) [33]	O; P; ER; PR; S; U	8 to 18 years	EG, n = 24, 13.17 ± 3.02, 62.5%
			CG, n = 22, 12,68 ± 3.00, 90.9%
Elnaggar et al. (2016) [27]	P (with isolated bilateral knee osteoarthritis)	N/A 9.7 (1.5) 10.1 (1.2)	EG, n = 15, 10.1 ± 1.2, N/A
			CG, n = 15, 9.7 ± 1.5, N/A,
Epps et al. (2005) [25]	O (extended or persistent); P; ER; PR; S	4 to 19 years.	EG, n = 39, 11 max 4-19, 49%
			CG, n = 39, 12 max 6-19, 62%
Baydogan et al. (2015) [36]	P (RF (+), RF (-)); O; PR	6 to 18 years	EG, n = 15, 10.00 ± 3.66, 73%
			CG, n = 15, 9.27 ± 1.43, 67%
Mendonca et al. (2013) [23]	O; P (RF (+)); S	8 to 18 years	EG, n = 25, 11.8 ± 3.4, 64%
			CG, n = 25, 11.0 ± 3.9, 64%
Tarakci et al. (2020) [28]	O; P; PR; S	5 to 17 years	EG, n = 43, 10.02 ± 3.44, 58%
			CG, n = 38, 10.82 ± 4.00, 50%
Azab et al. (2022) [24]	Not specified	10 to 14 years	EG, n = 20, 12.32 ± 1.67, 63.2%
			CG, n = 20, 11.56 ± 1.46, 77.8%
Sandstedt et al. (2013) [35]	O; P; ER; PR	9 to 21 years	EG, n = 33, 13.3 (8.8-19.9), 76%
			CG, n = 21, 14.9 (8.8-20.6), 81%

Pain Assessment

The Visual Analog Scale (VAS) and Numeric Rating Scale (NRS) were the most commonly utilized pain assessment tools across the studies [23-27,29-32,34,36]. Both scales employ a 10-centimeter line ranging from 0 to 10, with 10 representing maximum pain intensity. The key distinction is that the NRS includes numerical demarcations along the line, potentially offering greater precision than the unmarked VAS. The Childhood Health Assessment Questionnaire (CHAQ) was employed in several RCTs [28,33-35]. This instrument uses a 0-100 scale and may provide more granularity than the NRS, though potentially less sensitivity than the VAS. In CHAQ-based studies, the pain was sometimes referred to as the "discomfort index" or "physical pain." While the CHAQ assesses other variables, those were not pertinent to this analysis. Powell et al. uniquely utilized the Foot Function Index (FFI), which comprises nine items evaluating pain’s impact on activities [29]. Although not validated for pediatric populations, the FFI appeared suitable for their study design.

Online Treatments, Video Games, Low-Level Laser Therapy, and Orthoses

Table 2 summarizes the findings of RCTs investigating online interventions, video games, low-level laser therapy, and orthoses for pain alleviation in children with JIA. Two studies examined online interventions. Connelly et al. evaluated a 12-item online program focused on pain self-management and JIA education, comparing it to a control group receiving non-specific information without professional guidance [26]. The intervention group demonstrated a statistically significant reduction in pain (p < 0.05) as measured by the NRS [26]. Conversely, Armbrust et al. assessed an interactive, educational, and cognitive-behavioral online program termed Rheumates@Work (R@W) against usual care [31]. The R@W program did not yield significant improvements in pain as measured by the VAS, either at the initial assessment or at the one-year follow-up [31]. Regarding video game interventions, Arman et al. investigated the efficacy of the Kinect 360 console, which utilizes motion detection technology to provide real-time feedback [34]. Pain was assessed using the CHAQ for ADLs and the NRS during activities. Both the intervention group and the control group - which received occupational therapy for ADLs - demonstrated significant improvements from pre-treatment to post-treatment. However, no statistically significant differences were observed between the groups (p = 0.08) [34]. With respect to low-level laser therapy, a study by Elnaggar et al. evaluated its application on knee joints as an adjunctive treatment to physical exercise for JIA patients [32]. The low-level laser therapy group demonstrated statistically significant improvements in pain (p = 0.003), fatigue perception (p = 0.004), and functional status (p = 0.022) compared to the control group, which received exercises alone. However, no significant differences were observed in muscle performance between the groups [32]. Orthoses play a crucial role in the multidisciplinary management of joint-related pathologies. Two notable studies have investigated the efficacy of foot orthoses (FOs) in alleviating foot pain in patients with JIA [29,30]. FOs typically consist of rigid or semi-rigid supports extending across the entire sole and heel, designed to enhance stability and support during activities. Powell et al. compared three types of foot support: 1) custom-fitted semirigid FOs with shock-absorbing posts, 2) off-the-shelf flat neoprene shoe inserts, and 3) supportive athletic shoes featuring medial longitudinal arch support and shock-absorbing soles, worn without additional inserts [29]. The results revealed significant improvements in pain reduction for the custom-fitted semirigid FOs group, both when compared to baseline (p = 0.001) and between groups (p = 0.001), as measured by the VAS. Pain reduction was also observed over time in both the custom-fitted semirigid FOs group (p = 0.001) and the supportive athletic shoes group (p = 0.01). Furthermore, the FFI demonstrated a significant reduction in pain, with the custom-fitted semirigid FOs group showing superior results compared to other groups (p = 0.019) [29]. In a separate RCT, Coda et al. compared standard orthoses with individualized orthoses [30]. Using the VAS scale, they observed significant improvements in pain reduction for the individualized FO group at three- and six-months post-intervention (p < 0.01), with further improvements noted between the three- and six-month assessments (p < 0.05). However, the study did not include a direct comparison between the standard and individualized orthoses groups [29].

**Table 2:** Summary of RCTs evaluating online treatments, videogames, low-level laser therapy, and orthosis interventions for children with JIA VAS, Visual Analog Scale; R@W, Rheumates@Work; NRS, Numeric Rating Scale; EG, experimental group; CG, control group; TOAT, task-oriented activity training; DLC, daily living conditions; CHAQ, Child Health Assessment Questionnaire; FFI, Foot Function Index; FOs, foot orthoses; RCTs, randomized controlled trials; JIA, juvenile idiopathic arthritis

ONLINE TREATMENTS					
Armbrust et al. (2017) [31]	VAS	R@W program Cognitive behavioral program	14 weeks, four group sessions	No significant differences within or between groups.	
		Habitual care			
Conelly et al. (2019) [26]	NRS intensity; NRS pain interference	Online self-management program + brief monthly call with professionals	12 weeks, one item online per week	EG showed significant reduction in pain intensity and interference; no significant changes in CG.	
		Weekly website training + brief monthly call with professionals			
VIDEOGAMES					
Arman et al. (2019) [34]	CHAQ; NRS activity	TOAT with Xbox 360 Kinect	8 weeks 1-hour × 3 times/week	Significant improvements in CHAQ and NRS scores in both groups, no significant differences between groups.	
		TOAT in DLC			
LOW-LEVEL LASER THERAPY					
Elnaggar et al. (2022) [32]	VAS	Low-level laser therapy applied to knee joints + standard exercise program	3 months 40 min × 3 times/week	Significant improvements in both groups; EG showed superior improvement compared to CG.	
		Standard exercise program			
ORTHOSIS					
Powell et al. (2005) [29]	VAS; FFI	Custom-fitted semirigid FOs with shock-absorbing posts (group 1)	Three months, 24 h/day	Significant pain reduction in group 1; Group 1 showed superior improvement in VAS and FFI scores.	
		Off-the-shelf flat neoprene shoe inserts (group 2)			
		Supportive athletic shoes featuring medial longitudinal arch support and shock-absorbing soles, worn without additional inserts (group 3)			
Coda et al. (2014) [30]	VAS	Custom-fitted FOs	6 months, 24 h/day	Significant pain reduction from baseline to 3 and 6 months, favoring custom-fitted FOs.	
		Control FOs			

Hydrotherapy and Physical Exercise

Table 3 presents the results of studies investigating hydrotherapy and physical exercise. Hydrotherapy, an innovative and engaging treatment modality, was investigated in three RCTs as an intervention for JIA. Epps et al. compared hydrotherapy to land-based exercise groups, whereas Perez Ramirez et al. sought to evaluate the efficacy of Watsu therapy, which combines the therapeutic properties of water with the Japanese technique of Shiatsu, involving slow, controlled movements and massage, in comparison to traditional hydrotherapy [25,33]. Finally, Elnaggar et al. investigated a combination of aquatic exercises and interferential current, comparing it to conventional physiotherapy [27]. In the study by Epps et al., no changes were observed in the VAS; however, non-significant improvements in the CHQ were noted post-intervention and at follow-up, favoring the experimental group [25]. The land-based exercise group also demonstrated improvements post-treatment, albeit not statistically significant [25]. Using the CHAQ, Perez Ramirez et al. found significant post-treatment improvements in the Watsu therapy group (p = 0.031) compared to the control group [33]. Within-group analysis revealed significant enhancements after treatment (p = 0.007) for the experimental group. Elnaggar et al. reported significant differences between groups at one month (p = 0.043) and three months (p = 0.001) post-intervention, favoring the experimental group [27]. Within-group analysis showed significant improvements at each time point (p = 0.001) for both the experimental and control groups.

**Table 3:** Summary of RCTs evaluating hydrotherapy and land-based exercise interventions for children with JIA CG, control group; EG, experimental group; VAS, Visual Analog Scale; CHAQ, Child Health Assessment Questionnaire; CHQ, Child Health Questionnaire; NRS, Numeric Rating Scale; N/A, not applicable; RCTs, randomized controlled trials; JIA, juvenile idiopathic arthritis

HYDROTHERAPY					
Perez Ramirez et al. (2019) [33]	CHAQ	Watsu therapy	10 weeks 45 min × 1 time/week	Significant improvement from pre- to post-intervention (p = 0.031). Difference between EG and CG at post-intervention (p = 0.007) favoring EG. No significant changes at follow-up.	
		Conventional hydrotherapy			
Elnaggar et al. (2016) [27]	VAS	Combined resistive underwater exercises + interferential current therapy	Three months 45 min × 3 times/week	Significant differences at 1 month (p = 0.043) and 3 months (p = 0.001) favoring EG. Both interventions showed significant improvements over time (p = 0.001).	
		Traditional physical therapy			
Epps et al. (2005) [25]	VAS	Hydrotherapy and land physiotherapy	Two intensive weeks + 2 months	No significant change in VAS. CHQ scores improved at 2 months and significantly at 6 months in EG. No change in CG.	
		Land physiotherapy			
Baydogan et al. (2015) [36]	NRS-activity, resting, exercise	Balance-proprioceptive exercises	12 weeks 45 min × 3 times/week	Significant reduction in pain for both groups (p = 0.001). No significant difference between groups except for NRS-exercise (p = 0.011).	
		Strengthening exercises			
Mendonca et al. (2013) [23]	VAS	Pilates exercises	6 months 50 min × 2 times/week	No significant decrease in CG, significant reduction in EG (p = 0.0). Significant difference between baseline and 6-month follow-up for both CG and EG (p < 0.001), more pronounced in EG.	
		Conventional exercise program			
Tarakci et al. (2020) [28]	CHAQ (VAS)	Land-based home exercise + weekly change at hospital	12 weeks 20-45 min per week at hospital; 20-45 min × 3 times/week at home	Significant decreases in pain for both CG (p < 0.01) and EG (p < 0.001).	
		Waiting list			
Azab et al. (2022) [24]	VAS	Clinical pilates exercise	3 months 25-40 min × 3 times/week	Significant improvement in EG compared to CG (p < 0.001).	
		Conventional physical therapy			
Sandstedt et al. (2013) [35]	CHAQ/CHQ	Core and muscle strength program	12 weeks 3 times/week	No significant changes in pain in either group.	
		N/A			

Physical exercise encompasses a range of deliberate movements designed to enhance muscular tone, boost resilience to physical demands, and augment overall strength. Five RCTs investigated the use of physical exercise as a treatment modality for JIA. Among them, two specifically focused on physical exercise as the primary intervention. Specifically, Tarakci et al. utilized a waiting list control group design, whereas Sandstedt et al. compared the intervention to usual care [28,35]. In Tarakci et al.’s study, the experimental group performed a home-based physical activity program with weekly monitoring [28]. Post-intervention, both the control group (p < 0.01) and experimental group (p < 0.001) demonstrated significant reductions in pain. However, between-group comparisons on the VAS revealed no statistically significant differences [28]. Sandstedt et al. found no significant changes in CHAQ scores for either group at any time point [35]. However, it is important to note that physical exercise interventions can vary considerably in their specific modalities and may be combined with other therapeutic approaches. Two RCTs investigated the efficacy of Pilates exercises in treating JIA. Mendonca et al. compared Pilates to conventional physical exercise, while Azab et al. evaluated Pilates against traditional physiotherapy [23,24]. Pilates is distinguished from standard physical exercise by its emphasis on engaging deep muscles and incorporating breathing techniques. Mendonca et al. utilized the VAS within the CHAQ to measure pain [23]. Both the Pilates and physical exercise groups demonstrated significant pain reduction after treatment (p < 0.001). However, the CHAQ scores revealed a significant difference between the control and experimental groups after intervention, favoring the Pilates group (p < 0.0001) [23]. Similarly, Azab et al. reported a significant improvement in pain as measured by the VAS (p < 0.001) in favor of the Pilates group compared to conventional physiotherapy [24]. In a separate study, Baydogan et al. compared two distinct exercise modalities: proprioception-focused exercises (experimental group) and muscle-strengthening exercises (control group) [36]. Both groups showed significant improvements in pain post-intervention as measured by the NRS (p = 0.001). However, the between-group analysis revealed that only pain during exercise demonstrated a significant improvement favoring the proprioception group (p = 0.011). No significant differences were observed between groups for pain at rest (p = 0.502) or during activity (p = 0.672).

Meta-analysis

**Figure 2:**
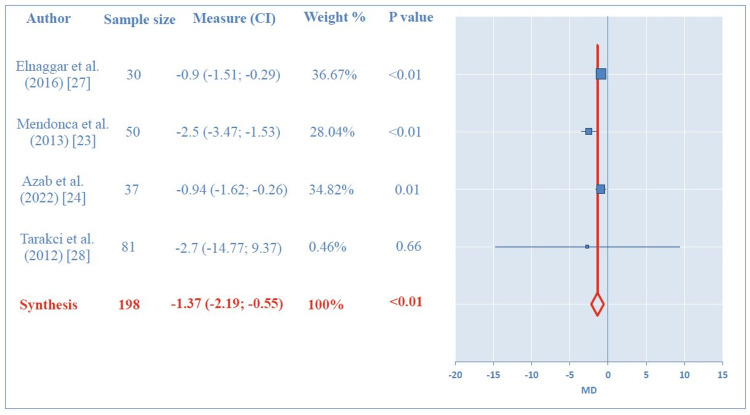
Forest plots comparing the effects of exercise interventions versus control on pain intensity in JIA MD, mean difference; CI, confidence interval; JIA, juvenile idiopathic arthritis

A meta-analysis was conducted to evaluate the effects of physical exercise interventions on chronic pain in patients with JIA. The analysis included data from four RCTs with a total sample size of 198 patients [23,24,27,28]. The interventions examined included Pilates and strength training, compared to control conditions such as waitlist, combined strength and aerobic exercise, or aquatic strength training. The pooled analysis revealed a significant beneficial effect of exercise interventions on chronic pain (mean difference (MD) = -1.37, 95% CI = -2.19 to -0.55, p < 0.01). No evidence of publication bias was detected using Begg’s test (p = 0.17). However, significant heterogeneity was observed among the RCTs (Q = 8; I2 = 65%, p = 0.040; Figure 2). Subgroup analyses further corroborated the beneficial effects of physical exercise on chronic pain in JIA. When comparing physical exercise interventions (e.g., Pilates and aquatic strength training) to conventional therapy (comprising flexibility, aerobic, and strength training combined), a pooled analysis of three studies (n = 117) revealed significant benefits (MD= -1.37, 95% CI = -2.25 to -0.5, p < 0.01) [23,24,27]. This analysis showed no evidence of publication bias (Begg’s test, p=0.12) but demonstrated significant heterogeneity among studies (Q = 8.42; I2 = 76%, p = 0.01). Similarly, when evaluating physical exercise (Pilates or strength training) against control conditions (including waiting list, flexibility or strength training, and combined flexibility and aerobic exercise), a pooled analysis of three studies (n = 168) also indicated significant benefits (MD = -1.69, 95% CI = -3.09 to -0.29, p = 0.02) [23,24,28]. This analysis likewise showed no evidence of publication bias (Begg’s test, p = 0.6) but exhibited significant heterogeneity among studies (Q = 6.69; I2 = 70%, p = 0.04).

Discussion

In this systematic review and meta-analysis of RCTs, we identified several conservative, non-pharmacological interventions for managing chronic pain in patients with JIA. The investigated approaches included online interventions, video games, low-level laser therapy, orthotic devices, hydrotherapy, and physical exercise. These modalities demonstrated varying degrees of efficacy in pain reduction. Notably, our meta-analytic findings indicate that structured physical exercise interventions may lead to statistically significant reductions in chronic pain intensity among patients with JIA. This observation highlights the potential of exercise-based therapies as a promising approach to pain management in this clinical population.

Non-pharmacological conservative interventions comprise a wide spectrum of therapeutic modalities, exhibiting varying levels of effectiveness in alleviating pain and enhancing functional capacity. In the context of JIA, emerging evidence suggests potential benefits from digital interventions, including online treatments and gamified programs, for pain management in children and adolescents, and the effects can last up to one year [26,31]. While video game-based interventions have shown encouraging results, the current evidence base remains limited [34]. However, the incorporation of gamification elements in pain management programs may enhance patient engagement and treatment adherence, presenting an innovative avenue worthy of additional exploration. Conversely, the use of orthoses in pediatric patients with JIA has been extensively researched, with evidence suggesting that well-fitted, semi-rigid devices are most effective for pain management [29,30]. Hydrotherapy, particularly the Watsu technique, has demonstrated significant efficacy in alleviating pain in JIA [33]. Furthermore, combining aquatic exercises with interferential current therapy has yielded a notable pain reduction [27]. However, isolated hydrotherapy interventions have not demonstrated significant pain improvement [25]. This highlights the importance of following a carefully structured aquatic therapy protocol to optimize pain management for patients with JIA. Collectively, these results indicate that orthoses have accumulated more convincing evidence. In contrast, the efficacy of other interventions such as online treatments, gamified programs, and hydrotherapy requires further investigation for pain management in JIA. Additionally, the effectiveness of low-level laser therapy as an adjunct to conventional physiotherapy remains inconclusive [32].

Our meta-analysis revealed a significant beneficial effect of exercise interventions on chronic pain in JIA. The underlying pathophysiological mechanisms for this observation are likely multifaceted. Resistance exercise has been shown to modulate immune function and optimize the balance between pro-inflammatory and anti-inflammatory signaling pathways, ultimately reducing pain perception [37]. The deep breathing associated with Pilates increases intra-abdominal pressure, potentially stabilizing the spine and reducing compressive forces [38]. Additionally, improved oxygenation and circulation to muscles resulting from Pilates breathing techniques, along with enhanced joint mobility may contribute to pain alleviation [39,40]. Aerobic exercise may also play a role in pain reduction through increased endorphin release, although none of the RCTs included in our systematic review employed interventions based solely on aerobic exercise [41]. Combining strength and aerobic exercises in a comprehensive program may produce synergistic effects [24,27,32]. Such strategies can improve both physical and emotional aspects of quality of life, potentially altering pain perception. Regarding the optimal duration of intervention, current evidence supports recommending a three-month program comprising two to three sessions (20-45 minutes each) per week [28]. However, future research should investigate whether physical exercise interventions for pain management in JIA should be further individualized beyond standard protocols [42]. Such investigations should consider potential confounding variables, including concomitant opioid use, and emphasize a multidisciplinary approach to treatment [43]. To enhance the effectiveness of these interventions, researchers should explore the integration of comprehensive pain education programs and the development of age-appropriate protocols specifically tailored for children and adolescents. These research directions hold significant promise for improving pain-related outcomes in JIA.

This review has two principal limitations that should be acknowledged. Although all included studies focused on polyarticular JIA, the absence of subgroup analyses raises questions about the generalizability of the findings to other JIA subtypes [30]. Additionally, all the studies utilized the ILAR criteria for diagnosing JIA, which define the onset of the disease before the age of 16. While this ensures homogeneity across the included research, it limits the scope of our findings, as patients diagnosed after the age of 16 under the broader PRINTO criteria were not considered. Despite these concerns, the findings of this review can be effectively integrated into clinical practice by physical therapists and healthcare professionals treating this patient population.

## Conclusions

This systematic review and meta-analysis highlight the critical importance of adopting a holistic approach to pain management in children and adolescents with JIA. Specifically, the integration of structured physical exercise programs into the multidisciplinary care framework for managing JIA-related chronic pain is strongly recommended. These exercise regimens should be sustained for a minimum of three months, with sessions occurring two to three times per week, each lasting approximately 45 minutes. This approach can potentially enhance the overall quality of life by significantly reducing pain intensity, thereby improving functional ability and enhancing the emotional and psychological well-being of children and adolescents with JIA.

## References

[1] Murray GM (2021). Advancing the treatment of juvenile idiopathic arthritis. Lancet.

[2] Martini A, Ravelli A, Avcin T (2019). Toward new classification criteria for juvenile idiopathic arthritis: first steps, Pediatric Rheumatology international trials Organization International consensus. J Rheumatol.

[3] Petty RE, Southwood TR, Manners P (2004). International League of Associations for Rheumatology classification of juvenile idiopathic arthritis: second revision, Edmonton, 2001. J Rheumatol.

[4] Chen K, Zeng H, Togizbayev G, Martini A, Zeng H (2023). New classification criteria for juvenile idiopathic arthritis. Int J Rheum Dis.

[5] Zaripova LN, Midgley A, Christmas SE, Beresford MW, Baildam EM, Oldershaw RA (2021). Juvenile idiopathic arthritis: from aetiopathogenesis to therapeutic approaches. Pediatr Rheumatol Online J.

[6] Dave M, Rankin J, Pearce M, Foster HE (2020). Global prevalence estimates of three chronic musculoskeletal conditions: club foot, juvenile idiopathic arthritis and juvenile systemic lupus erythematosus. Pediatr Rheumatol Online J.

[7] Martini A, Lovell DJ (2010). Juvenile idiopathic arthritis: state of the art and future perspectives. Ann Rheum Dis.

[8] Mellins ED, Macaubas C, Grom AA (2011). Pathogenesis of systemic juvenile idiopathic arthritis: some answers, more questions. Nat Rev Rheumatol.

[9] Verwoerd A, Ter Haar NM, de Roock S, Vastert SJ, Bogaert D (2016). The human microbiome and juvenile idiopathic arthritis. Pediatr Rheumatol Online J.

[10] Martini A, Lovell DJ, Albani S, Brunner HI, Hyrich KL, Thompson SD, Ruperto N (2022). Juvenile idiopathic arthritis. Nat Rev Dis Primers.

[11] Stoll ML, Cron RQ (2014). Treatment of juvenile idiopathic arthritis: a revolution in care. Pediatr Rheumatol Online J.

[12] Hughes DA, Culeddu G, Plumpton CO (2019). Cost-effectiveness analysis of adalimumab for the treatment of uveitis associated with juvenile idiopathic arthritis. Ophthalmology.

[13] Jacobson JL, Pham JT (2018). Juvenile idiopathic arthritis: a focus on pharmacologic management. J Pediatr Health Care.

[14] Boonstra AM, Reneman MF, Waaksma BR, Schiphorst Preuper HR, Stewart RE (2015). Predictors of multidisciplinary treatment outcome in patients with chronic musculoskeletal pain. Disabil Rehabil.

[15] Li WH, Chung JO, Ho KY, Kwok BM (2016). Play interventions to reduce anxiety and negative emotions in hospitalized children. BMC Pediatr.

[16] Skúladóttir H, Sveinsdottir H, Holden JE, Gunnarsdóttir TJ, Halldorsdottir S, Björnsdottir A (2021). Pain, Sleep, and Health-Related Quality of Life after Multidisciplinary Intervention for Chronic Pain. Int J Environ Res Public Health.

[17] Heinrich-Rohr M, Moenkemoeller K, Niewerth M (2021). Consumer perspective on healthcare services for juvenile idiopathic arthritis: results of a multicentre JIA inception cohort study. Clin Exp Rheumatol.

[18] Page MJ, McKenzie JE, Bossuyt PM (2021). The PRISMA 2020 statement: an updated guideline for reporting systematic reviews. BMJ.

[19] Maher CG, Sherrington C, Herbert RD, Moseley AM, Elkins M (2003). Reliability of the PEDro scale for rating quality of randomized controlled trials. Phys Ther.

[20] Dettori JR, Norvell DC, Chapman JR (2022). Fixed-effect vs random-effects models for meta-analysis: 3 points to consider. Global Spine J.

[21] Borenstein M, Hedges LV, Higgins JP, Rothstein HR (2010). A basic introduction to fixed-effect and random-effects models for meta-analysis. Res Synth Methods.

[22] Bax L, Yu LM, Ikeda N, Tsuruta H, Moons KG (2006). Development and validation of MIX: comprehensive free software for meta-analysis of causal research data. BMC Med Res Methodol.

[23] Mendonça TM, Terreri MT, Silva CH, Neto MB, Pinto RM, Natour J, Len CA (2013). Effects of Pilates exercises on health-related quality of life in individuals with juvenile idiopathic arthritis. Arch Phys Med Rehabil.

[24] Azab AR, Kamel FH, Basha MA (2022). Impact of clinical Pilates exercise on pain, cardiorespiratory fitness, functional ability, and quality of life in children with polyarticular juvenile idiopathic arthritis. Int J Environ Res Public Health.

[25] Epps H, Ginnelly L, Utley M, Southwood T, Gallivan S, Sculpher M, Woo P (2005). Is hydrotherapy cost-effective? A randomised controlled trial of combined hydrotherapy programmes compared with physiotherapy land techniques in children with juvenile idiopathic arthritis. Health Technol Assess.

[26] Connelly M, Schanberg LE, Ardoin S (2019). Multisite randomized clinical trial evaluating an online self-management program for adolescents with juvenile idiopathic arthritis. J Pediatr Psychol.

[27] Elnaggar RK, Elshafey MA (2016). Effects of combined resistive underwater exercises and interferential current therapy in patients with juvenile idiopathic arthritis: a randomized controlled trial. Am J Phys Med Rehabil.

[28] Tarakci E, Yeldan I, Baydogan SN, Olgar S, Kasapcopur O (2012). Efficacy of a land-based home exercise programme for patients with juvenile idiopathic arthritis: a randomized, controlled, single-blind study. J Rehabil Med.

[29] Powell M, Seid M, Szer IS (2005). Efficacy of custom foot orthotics in improving pain and functional status in children with juvenile idiopathic arthritis: a randomized trial. J Rheumatol.

[30] Coda A, Fowlie PW, Davidson JE, Walsh J, Carline T, Santos D (2014). Foot orthoses in children with juvenile idiopathic arthritis: a randomised controlled trial. Arch Dis Child.

[31] Armbrust W, Bos GJ, Wulffraat NM (2017). Internet program for physical activity and exercise capacity in children with juvenile idiopathic arthritis: a multicenter randomized controlled trial. Arthritis Care Res (Hoboken).

[32] Elnaggar RK, Mahmoud WS, Abdelbasset WK, Alqahtani BA, Alrawaili SM, Elfakharany MS (2022). Low-energy laser therapy application on knee joints as an auxiliary treatment in patients with polyarticular juvenile idiopathic arthritis: a dual-arm randomized clinical trial. Lasers Med Sci.

[33] Pérez Ramírez N, Nahuelhual Cares P, San Martín Peñailillo P (2019). Effectiveness of Watsu therapy in patients with juvenile idiopathic arthritis. A parallel, randomized, controlled and single-blind clinical trial (Spanish). Rev Chil Pediatr.

[34] Arman N, Tarakci E, Tarakci D, Kasapcopur O (2019). Effects of video games-based task-oriented activity training (Xbox 360 Kinect) on activity performance and participation in patients with juvenile idiopathic arthritis: a randomized clinical trial. Am J Phys Med Rehabil.

[35] Sandstedt E, Fasth A, Eek MN, Beckung E (2013). Muscle strength, physical fitness and well-being in children and adolescents with juvenile idiopathic arthritis and the effect of an exercise programme: a randomized controlled trial. Pediatr Rheumatol Online J.

[36] Baydogan SN, Tarakci E, Kasapcopur O (2015). Effect of strengthening versus balance-proprioceptive exercises on lower extremity function in patients with juvenile idiopathic arthritis: a randomized, single-blind clinical trial. Am J Phys Med Rehabil.

[37] Rochette E, Duché P, Merlin E (2015). Juvenile idiopathic arthritis and physical activity: possible inflammatory and immune modulation and tracks for interventions in young populations. Autoimmun Rev.

[38] Campos de Oliveira L, Gonçalves de Oliveira R, Pires-Oliveira DA (2015). Effects of Pilates on muscle strength, postural balance and quality of life of older adults: a randomized, controlled, clinical trial. J Phys Ther Sci.

[39] Fernández-Rodríguez R, Álvarez-Bueno C, Cavero-Redondo I (2022). Best exercise options for reducing pain and disability in adults with chronic low back pain: Pilates, Strength, Core-based, and mind-body. A network meta-analysis. J Orthop Sports Phys Ther.

[40] Laroche D, Pozzo T, Ornetti P, Tavernier C, Maillefert JF (2006). Effects of loss of metatarsophalangeal joint mobility on gait in rheumatoid arthritis patients. Rheumatology (Oxford).

[41] Rice D, Nijs J, Kosek E (2019). Exercise-induced hypoalgesia in pain-free and chronic pain populations: state of the art and future directions. J Pain.

[42] Bull FC, Al-Ansari SS, Biddle S (2020). World Health Organization 2020 guidelines on physical activity and sedentary behaviour. Br J Sports Med.

[43] Weiss JE, Luca NJ, Boneparth A, Stinson J (2014). Assessment and management of pain in juvenile idiopathic arthritis. Paediatr Drugs.

